# Supplementary material for: Navigating tenses in Bengali sentences: A stacked ensemble model for enhanced prediction
Source: PLoS One. 2025 Aug 21;20(8):e0330186. doi: 10.1371/journal.pone.0330186 (PMC12370126; doi:10.1371/journal.pone.0330186)
Supplement: Attachment — Submitted filename: Response to Reviewers_PONE-D-24-57203.pdf [file pone.0330186.s001.pdf]

Title: "Navigating Tenses in Bengali Sentences: A Stacked Ensemble Model for Enhanced Prediction" (Submission ID: PONE-D-24-57203)

Response to Reviewer 1: (Color code= Red)

#Point 1:

Abstract needs complete rewrite. Add concrete info on model type, dataset used, accuracy achieved, and contribution.

Author Response:

Thank you for your valuable and constructive feedback. In response to your comment, we have completely revised the abstract to clearly articulate the scope, methodology, and contributions of our work. The updated abstract now specifies the NLP task of Bengali tense classification and its relevance to downstream applications. We describe the construction of a novel dataset, "BengaliTenseCorpus," comprising 13,500 manually labeled sentences across three tense categories. Additionally, we detail our proposed stacked ensemble model integrating five base classifiers (Random Forest, SVM, XGBoost, LSTM, and GRU) with a neural network meta-learner, and report the achieved test accuracy of 85%. We also highlight the novelty of combining machine learning and deep learning techniques for this task and outline future directions. These additions ensure the abstract conveys the model type, dataset used, accuracy obtained, and the overall contribution of our work, in line with your suggestion.

Author Action:

Based on the reviewer's comments, we have updated our **Abstract Section** to meet the reviewer's concerns.

#Point 2:

In intro, Clearly state the objective. Literature is not well synthesized. Review of recent deep learning NLP works for Bengali is missing.

Author Response:

Thank you for your thoughtful feedback. In response, we have revised the introduction to explicitly state the main objective of the study as developing a robust stacked ensemble model for automatic tense classification in Bengali sentences and its significance in the context of Bangla NLP. Additionally, we have strengthened the literature review by synthesizing existing works more cohesively and incorporating several recent studies that apply deep learning techniques to Bengali language tasks, such as classification, prediction, and detection. These additions provide a clearer context for our contribution and ensure that the background is both comprehensive and current. We believe these revisions directly address the reviewer's concerns and enhance the clarity and relevance of the introduction.

Author Action:

Based on the reviewer's comments, we have updated our **Introduction Section and Literature Review Section** to meet the reviewer's concerns.

#Point 3:
No clear novelty. Stacking models is not new. Authors must explicitly say: “Our novel contribution is…” and justify how this differs from prior Bengali NLP work.

Author Response:
Thank you for your kind remarks. There is little to no work present in the predictive model to predict the tense of Bangla text. The sole reason for this problem is the lack of a valid dataset. Our novel contribution is to create a meaningful dataset for Bangla tense prediction and create a predictive model around it

Author Action:
We have included our novel contribution statement at the end of the introduction section according to the reviewer's suggestions.

#Point 4:
There is no detail on train test split, feature preprocessing, or hyperparameter settings.

Author Response:
Thank you for your valuable feedback.

Author Action:
With the renamed subsection of "Model Selection and Hyper Parameters," all information pertaining to the train-test split and hyper hyperparameter setup is added to the methodology section. Likewise, details regarding feature processing are included in the methodology part of the paragraph titled "Data Pre-processing".

#Point 5:
Authors mention base learners but don’t explain architecture or why those were chosen. Provide diagrams or a clear pipeline.

Author Response:
We selected base models based on their empirical performance during cross-validation. Each model demonstrated strong predictive ability individually, which makes them suitable candidates for contributing diverse and accurate predictions in the ensemble. Our ensemble model consists of machine learning models and deep learning models. These models use different learning biases. This diversity is essential in stacking to ensure the meta-learner can generalize well across prediction errors. A clear pipeline is presented in Figure 10.

Author Action:
A brief discussion of our preferred models while creating the ensemble model is given in “Our Model” subsection.

#Point 6:
What metric is used for “highest accuracy” (line 91)? Is it F1? Precision?

Author Response:
Thank you for your feedback. The accuracy score of such a model is referred to as the highest accuracy. For that model, the F1 score, precision, and recall were all 0.989.

Author Action:
We have rewritten the sentence accordingly.

#Point 7:
Add a Limitation Paragraph.

Author Response:
We appreciate reviewers' valuable insight.

Author Action:
A paragraph has been added at the end of the conclusion section to discuss the limitations and future work.

#Point 8:
Replace sentences like “paves the way for further research” (line 108) with real actionable outcomes. Avoid over generalization. Needs professional proofreading.

Author Response:
In response, we have revised overgeneralized statements such as “paves the way for further research” (line 108) and replaced them with specific, actionable outcomes related to dataset expansion, transformer-based model integration, and tense morphology conversion. Additionally, the entire manuscript has been carefully proofread and refined to improve clarity, precision, and professionalism in language. We believe these changes enhance both the quality and specificity of the manuscript.

Author Action:
Based on the reviewer’s comments, we have replaced the statement with constructive outcome.

#Point 9:
Read these studies to further enrich your study, (https://doi.org/10.1371/journal.pone.0312395) Supports your paper's socioeconomic perspective, especially how economic indicators shape inequality using ML. (https://doi.org/10.1371/ journal.pone.0303883) Demonstrates use of ML with economic indicators in the Sri Lankan context, aligning with your economic modeling. (https://doi.org/10.1371/journal.pone.0282847) Reinforces your use of hybrid models like ANFIS and soft computing in Sri Lankan environmental data modeling.

Author Response:
Thank you for recommending these valuable studies. We have reviewed all three articles and incorporated relevant insights into the manuscript to strengthen the socioeconomic and methodological context of our work. Specifically, we referenced the use of machine learning in economic inequality modeling and hybrid approaches like ANFIS in environmental data analysis, particularly in low-resource contexts similar to ours. These additions have enriched the background and discussion sections and helped to better position our contribution within existing literature.

Author Action:
Based on the reviewer’s comments, we have updated the literature review.

# Response to Reviewer 2: (Color code= Green)

#Point 1:

You mention broad goals around improving accuracy and language modeling for Bengali, but you never define the specific task (e.g., classification? sentiment? named entity recognition?) in clear terms at the beginning. Define the target NLP task explicitly and connect it to your broader motivation, whether it's education, policy, or social impact in Bengali speaking regions.

Author Response:

In response, we have revised the introduction to clearly define the target NLP task as *automatic tense classification* in Bengali text. We have explicitly stated this task early in the introduction and connected it to broader motivations such as improving educational tools, supporting grammar correction, and enabling more inclusive and effective NLP technologies for Bengali-speaking communities. This clarification helps to better frame the scope and societal relevance of our work.

Author Action:

Updated the introduction to explicitly define the target NLP task as automatic tense classification and linked it to broader motivations such as educational support, grammar correction, and enhancing NLP applications for Bengali-speaking communities.

#Point 2:

You claim your stacked model performs better, but there's no comparison with a baseline model (e.g., logistic regression, SVM, or even a single deep model). This makes it impossible to assess whether the stacking architecture adds real value. Include results from simpler models to show performance gains.

Author Response:

We thank the reviewers for their valuable insights. We included a detailed comparison table of several machine learning models (6 models) and deep learning (3 models) models' performance in the "Results and Discussion" section, with accuracy, recall, and F-1 scores. In every case, our proposed stacking ensemble model outperformed all the machine learning and deep learning models.

Author Action:

We included a paragraph stating the information in the "Proposed Combined Model" section.

#Point 3:

There's no mention of whether the dataset is balanced. If your dataset has skewed classes (which is common in real world Bengali text), then accuracy alone is misleading. Did you use class weights, oversampling, or SMOTE? This should be discussed and reflected in metrics like F1 or recall per class.

Author Response:

Thank you for this valuable observation. Our dataset was balanced. For that reason, we didn't require any oversampling techniques. The support value that reflects the per-class instance is present in every table for every model in the "Results and Discussion" section.

Author Response:

We have also included the dataset information about balancing in "Dataset Collection and Properties" subsection.

#Point 4:

Beyond noting the language, you don't discuss the linguistic characteristics of Bengali that make this NLP task uniquely challenging (e.g., rich morphology, low resource nature, ambiguous syntax). Add some language specific insights to justify why your model is suitable and necessary.

Author Response:

Thank you for this valuable observation. In response, we have revised the introduction to include a detailed discussion of Bengali's linguistic characteristics that make tense classification particularly challenging. Specifically, we address its rich inflectional morphology, low-resource nature, syntactic ambiguity, and the predominance of suffix-based tense marking. We also explain how our stacked ensemble model is well-suited to handle these complexities by combining deep learning for sequence modeling and machine learning for robust pattern recognition. These additions help clarify the necessity and relevance of our approach

Author Action:

We have Revised the introduction to include a detailed explanation of Bengali's linguistic features, such as its rich morphology, low-resource status, and syntactic ambiguity, and justified the suitability of the proposed stacked ensemble model in addressing these challenges.

#Point 5:

Given that your stacked model is complex, how do you explain or interpret its predictions? Even a brief mention of SHAP values, attention weights, or feature importance would increase trust and make the model more actionable for policymakers or users in the region.

Author Response:

We appreciate the reviewer's emphasis on model interpretability, particularly given the complexity of our stacked ensemble and the importance of actionable insights for policymakers. While SHAP analysis is challenging to apply directly to our current ensemble due to the heterogeneous nature

of the base models and their text processing pipelines, we agree that interpretability is critical for transparency and trust. In future work, we aim to incorporate token-level attribution methods such as SHAP.

Author Action:

Added as Future Work in the Conclusion section.

#Point 6:

Refer to this work, relevant to your socio contextual motivation of language based emotion/sentiment classification in noisy settings. DOI: 10.1038/s41598-024-67269-2

Author Response:

In response, we have tried to reach this paper. We found a similar paper titled "Customer Sentiment Recognition in Conversation Based on Contextual Semantic and Affective Interaction Information"

Author Action:

We have included the paper in Literature Review section

Again, thank you for your kind cooperation and consideration.

Best Regards,
Md. Nahid Hasan
Corresponding Author
